# Efficient Excitation of Micro/Nano Resonators and Their Higher Order Modes

**DOI:** 10.1038/s41598-018-36482-1

**Published:** 2019-01-22

**Authors:** N. Jaber, M. A. A. Hafiz, S. N. R. Kazmi, M. H. Hasan, F. Alsaleem, S. Ilyas, M. I. Younis

**Affiliations:** 1 Physical Sciences and Engineering Division, King Abdullah University of Science and Technology, Thuwal, 23955-6900 Saudi Arabia; 2 Durham School of Architectural Engineering and Construction, University of Nebraska Lincoln, Lincoln, Nebraska 68182-0816 USA

## Abstract

We demonstrate a simple and flexible technique to efficiently activate micro/nano-electromechanical systems (MEMS/NEMS) resonators at their fundamental and higher order vibration modes. The method is based on the utilization of the amplified voltage across an inductor, *L*, of an *LC* tank resonant circuit to actuate the MEMS/NEMS resonator. By matching the electrical and mechanical resonances, significant amplitude amplification is reported across the resonators terminals. We show experimentally amplitude amplification up to twelve times, which is demonstrated to efficiently excite several vibration modes of a microplate MEMS resonator and the fundamental mode of a NEMS resonator.

## Introduction

Electrostatically actuated MEMS/NEMS resonators have demonstrated great potential in wide range of applications, such as sensors^1–3^, communication devices^4,5^, logic gates^6–8^, and quantum measurements^9–12^. This can be attributed to their low power consumption, ease of fabrication, and compatibility with complementary metal-oxide-semiconductor (CMOS) fabrication processes^13,14^. However, these devices suffer from low signal-to-noise ratio due to the reduced capacitive area for actuation and detection. Also, exploiting higher order vibration modes of MEMS/NEMS resonant structures requires high voltages that cannot be attained using conventional function generators and power supplies. Sensitivity of a resonant sensor can be increased by shrinking their size; preferably to the nanometer range. However, this reduces the capacitive area available for actuation/detection, and dramatically increases the resonator stiffness, hence, the corresponding actuation voltages.

Different dynamical principles have been investigated to enhance the sensitivity of resonator-based sensors, such as bifurcations, jumps, instabilities (for example pull-in)^15–18^, higher order modes excitations^19,20^, and sub-harmonic and super-harmonic resonances^21^. However, these techniques require high actuation voltages, which often are above the standard range of conventional function generators and on-chip power supplies. Employing active electronics to amplify the signal increases the device footprint, power consumption, and cost. Moreover, amplifiers have limited gain at high frequencies. Parametric excitation has been explored to achieve mechanical amplification^22,23^. However, it requires special structural designs (to enable modulating the stiffness) and ultra-low damping conditions. Hence, it is of crucial importance to develop flexible, simple, and CMOS compatible techniques to boost the actuation force to excite MEMS/NEMS resonators at their fundamental and higher order modes.

Electrical resonant circuits composed of inductor (*L*) and capacitor (*C*) connected in series or parallel have been employed as sensors in wide range of applications including temperature monitoring^24^, chemical detection^25^, and pressure sensing^26^. The concept is based on tracking the shift in the electrical resonant frequency or impendence variation due to the change in a physical stimulus. Matching the electrical resonance frequency of an *LC* tank circuit, *f*_*LC*_, with the mechanical resonance frequency of a NEMS resonator, *f*_*m*_, has shown to enable the sensitive detection of the motional response of an array of NEMS resonators^27^, increase the efficiency of microwave detection^11,28^, and has also been proposed for energy harvesting^29,30^. The amplified voltage across the capacitor of an *LC* tank circuit has been exploited to measure the position of the microstructure, and amplify the on-chip available voltage^31^. It has also been used to stabilize the movable parallel plate beyond the traditional pull-in point^32^. In addition, in our previous work, we utilized the electrical resonance phenemenon to amplify the response of a single port MEMS structure^33^.

Electrical resonance occurs when the excitation frequency matches the electrical resonance frequency of the *LC* tank circuit at which the circuit reactance goes to zero. At this driving frequency, the current flowing in the circuit gets amplified, which leads to an amplified voltage across the individual components (capacitor, inductor) of the *LC* tank circuit. Thus, the device experiences the voltage amplification due to electrical resonance leading to large vibration amplitude, which is desirable for sensing, actuation, and energy harvesting. Although many studies have explored the *LC* tank circuits in wide range of applications, there is a lack of thorough understanding of the potential of this technique in activating MEMS and NEMS resonators and their higher order modes. To demonstrate the flexibility and effectiveness of this technique, we investigate its utilization in three different scenarios (case studies). In the first, we match the *LC* tank resonance frequency, *f*_*LC*_, with the different mechanical resonance frequencies *f*_*m*_ of a microplate resonator by tuning an external inductor *L*. In the second case study, we employ the mixed frequency excitation technique, at which the resonator is excited using a multifrequency signal composed of two AC sources, *v*_*AC*1_ and *v*_*AC*2_ of frequencies *f*_*1*_ and *f*_*2*_, respectively, superimposed to a DC voltage. Due to the quadratic nature of the electrostatic actuation, the frequency spectrum of the resulting signal contains the following six frequency components (*f*_*1*_, *f*_*2*_, *2f*_*1*_, *2f*_*2*_, *f*_*1*_ + *f*_*2*_, and *f*_*1*_ − *f*_*2*_). The *LC* resonance is matched with one of the excitation frequencies *f*_*1*_ = *f*_*LC*_, while the mechanical resonance is excited by the combination resonance *(f*_*1*_ ± *f*_*2*_*)* = *f*_*m*_ ^34,35^. In the third case study, we use the mixing scheme and demonstrate an efficient actuation of a nanobeam near its fundamental mode.

**Figure 1:**
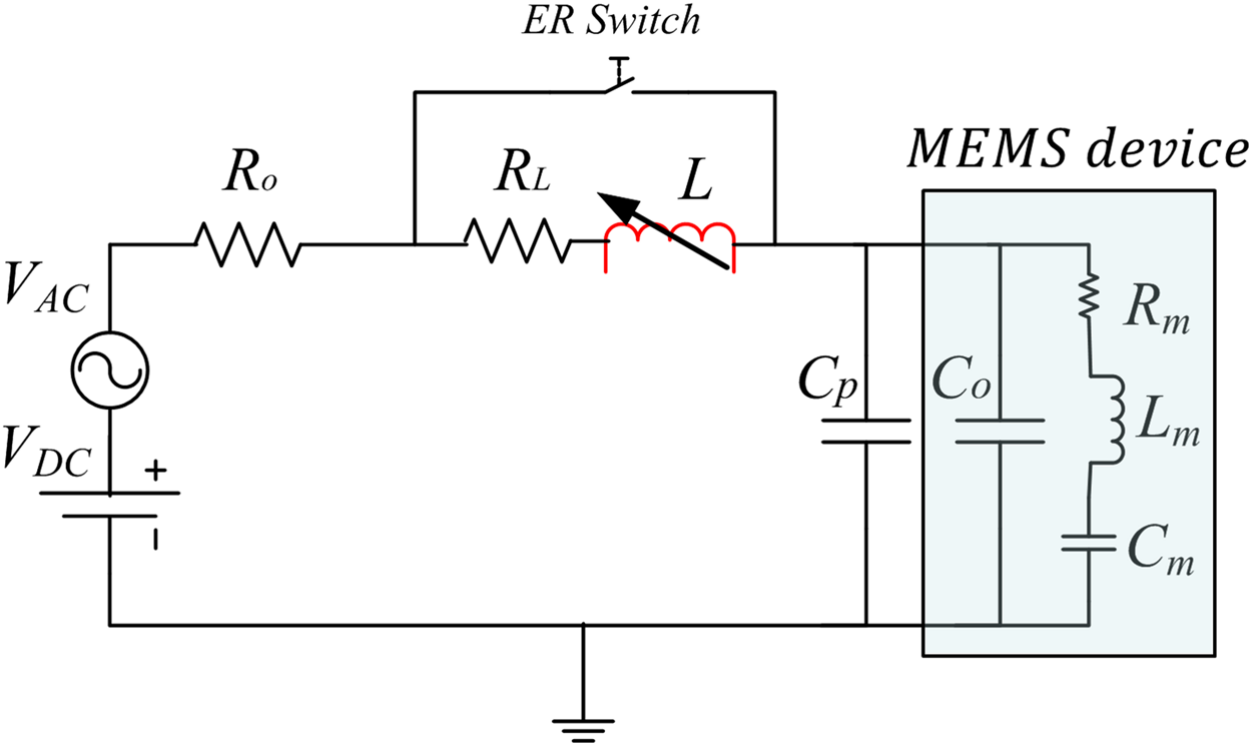
Schematic of the *LC* tank circuit showing the external tunable inductor connected in series with the device capacitance, *C*_*o*_. *C*_*p*_ represents the cables parasitic capacitance, *R*_*o*_ is the internal resistance of the different components in the circuit, and *R*_*L*_ is the inductor internal resistance. *R*_*m*_, *L*_*m*_, and *C*_*m*_ model the motional behavior of the resonator. Closing the ER switch will deactivate the electrical resonance.

As shown in Fig. 1, an external tunable inductor, *L*, and ER switch are connected in series with the MEMS device, to form the electrical resonance circuit. Closing the ER switch will deactivate the electrical resonance. The electrical resonance circuit characteristic equation is given as follow:

1 $$L\frac{{d}^{2}Q}{d{t}^{2}}+R\frac{dQ}{dt}+\frac{1}{C}Q={V}_{in}cos(2\pi ft)$$

where *R* is the total resistance in the circuit which composed from the internal resistances of the different componenets, *R*_*o*_, and the inductor internal resistance *R*_*L*_, *Q* is the electric charge, and *C* is the overall system capacitance composed of the device capacitance, *C*_*o*_, and the cables parasitic capacitance, *C*_*p*_. By solving Eq. (1), the steady state voltage across the device, *V*_*C*_, can be obtained using Eq. (2)

2 $${V}_{C}\approx \frac{{V}_{in}}{\sqrt{{(2\pi fRC)}^{2}+{({(2\pi f)}^{2}LC-1)}^{2}}}$$

When the input frequency, *f*, matches the electrical resonant frequency,${f}_{LC}=\frac{1}{2\pi \sqrt{LC}}$, the reactance of the inductor and capacitor cancels each other, hence maximizing the current flowing in the circuit. At resonance, the voltage across the device is increased by a factor of *Q*_*LC*_ as shown in Eq. (3)

3 $${V}_{C}\approx \frac{{V}_{in}}{(2\pi fRC)}={Q}_{LC}\times {V}_{in}$$

where *Q*_*LC*_ is the quality factor of the electrical resonance circuit defined as

4 $${Q}_{LC}=\frac{1}{R}\sqrt{\frac{L}{C}}$$

## Materials and Methods

### Fabrication

**Figure 2:**
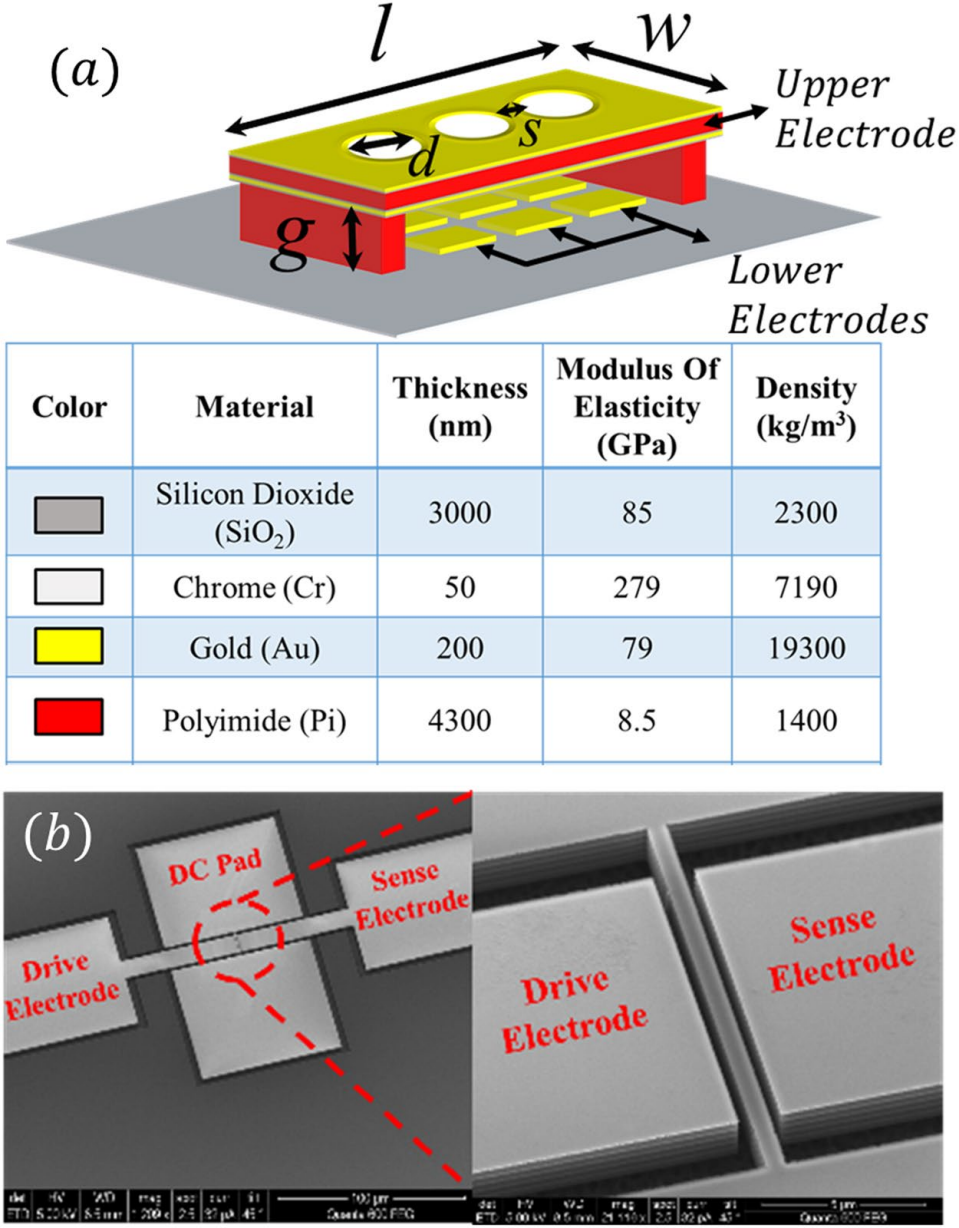
(**a**) Schematic of the microplate with the partial lower electrode configuration utilized in the first and second case studies, showing the material types, properties, and thicknesses. (**b**) An SEM image of the nanobeam resonator used in the third case study.

Figure 2(a) shows a schematic of the fabricated clamped-clamped free-free microplate of length (*l*) *400* *µm*, width (*w*) *150* *µm*, holes diameter (*d*) *60* *µm*, and spacing (*S*) 55 *µm*. The perforations reduce the time required to fully release the microplate and decrease the influence of squeeze film damping. The microplate is composed of polyimide layer of thickness *4.2* *µm* coated from top and bottom with Chrome and Gold layers of *50* *nm* and *200* *nm* thicknesses, respectively. The lower electrode is divided into six parts; each one can be separately accessed for electrical connection. The proposed design facilitates the excitation of higher order modes by connecting different portions of the lower electrode to excite the desired mode shape^19^. The upper and lower electrodes are separated by a 3.3 *µm* air gap. The detail of the fabrication process can be found in^36^. When the two electrodes are connected to external excitation voltage, the microplate vibrates in the out of plane direction.

Figure 2b shows an SEM image of the nanobeam resonator of length (*l*) 14.7 *µm*, width (*w*) *680* *nm*, thickness (*t*) 1.5 *µm*, and gap (*g*) 360 *nm*. The detail of the nanobeam fabrication process can be found in^37^.

### Experimental setup

**Figure 3:**
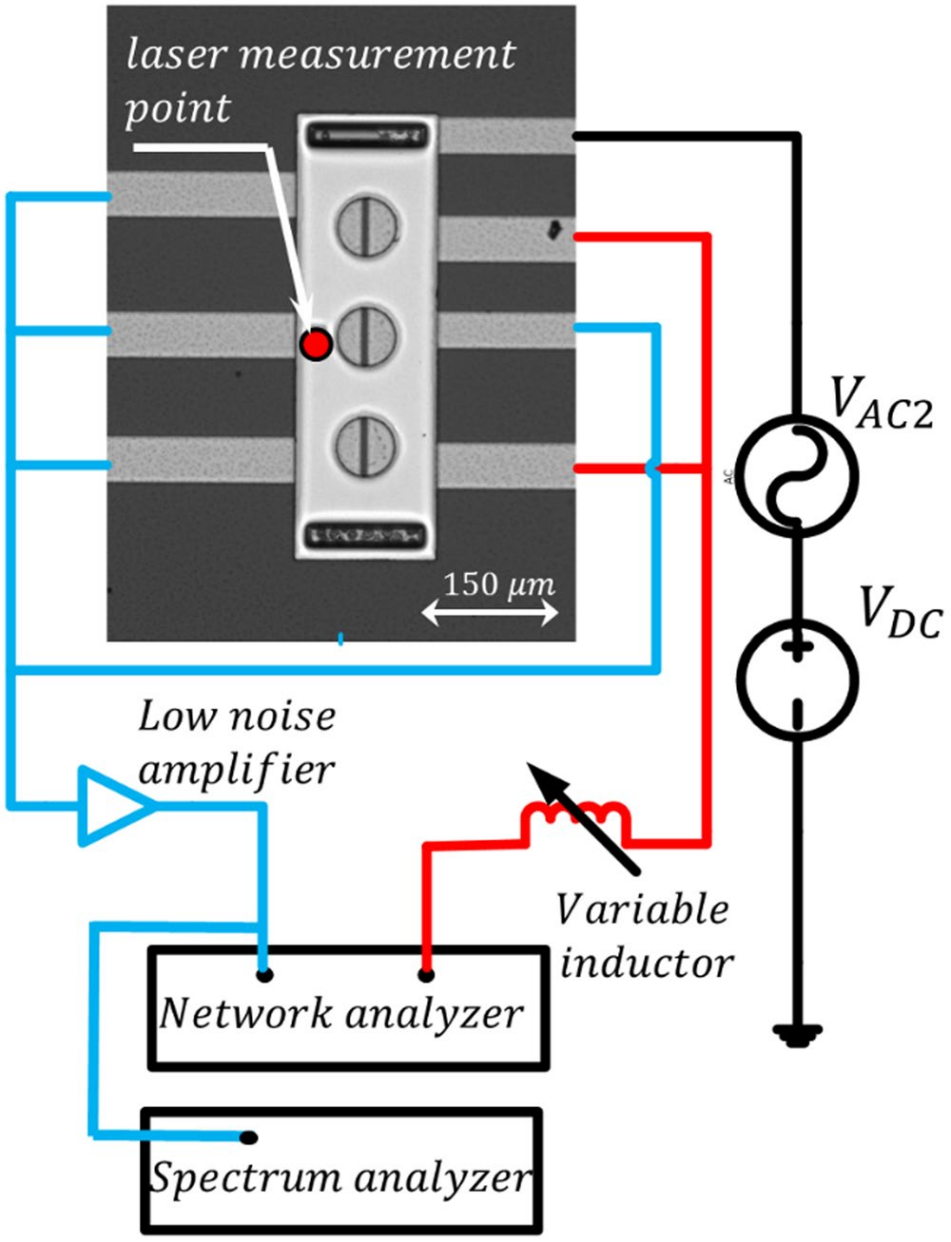
The characterization setup showing the microplate, network analyzer, low noise amplifier, DC source, spectrum analyzer, and the external tunable inductor.

To demonstrate the flexibility and simplicity of the proposed actuation technique, we utilize different characterization schemes, direct excitation^33^ and frequency mixing^4^. In the direct excitation scheme, we utilize a network analyzer (Agilent E5071C) to actuate the resonator with an AC signal, *v*_*AC*_ = *V*_*AC*_ cos 2π*ft*, connected to an inductor *L*, which is connected to the drive electrodes. For the microplate, we utilize two electrodes to actuate the resonator and other four electrodes to sense the output motional current as depicted in Fig. 3. This configuration allows the excitation of the first out of plane mode *ω*_*11*_ as well as the higher order mode *ω*_*32*_. Using a low noise amplifier (LNA), we amplify the output signal before connecting it to the input port of the network analyzer for the transmission signal measurement. Also, a laser Doppler vibrometer is used to measure the amplitude of vibration of the microplate due to different level of voltages and input frequencies. Using LabVIEW, the recorded signal is post-processed to generate the frequency response curves. The microplate is biased with a DC voltage *V*_*DC*_ connected to the upper electrode and tested at vacuum condition, *P* = *60 mTorr*.

In the mixing technique, in addition to the tools utilized in the direct excitation technique, we utilize a bias tee to connect an additional AC source, *v*_*AC*2_ = *V*_*AC*2_ cos 2π*f*_2_*t*, with the bias DC voltage, which is then connected to the upper electrode. Also, a spectrum analyzer is used to record the generated output signal as shown in Fig. 3. In addition, to characterize the nanobeam, we employ a probe station, ST-500 JANIS, under controlled pressure and temperature conditions.

## Results

### Case 1

**Figure 4:**
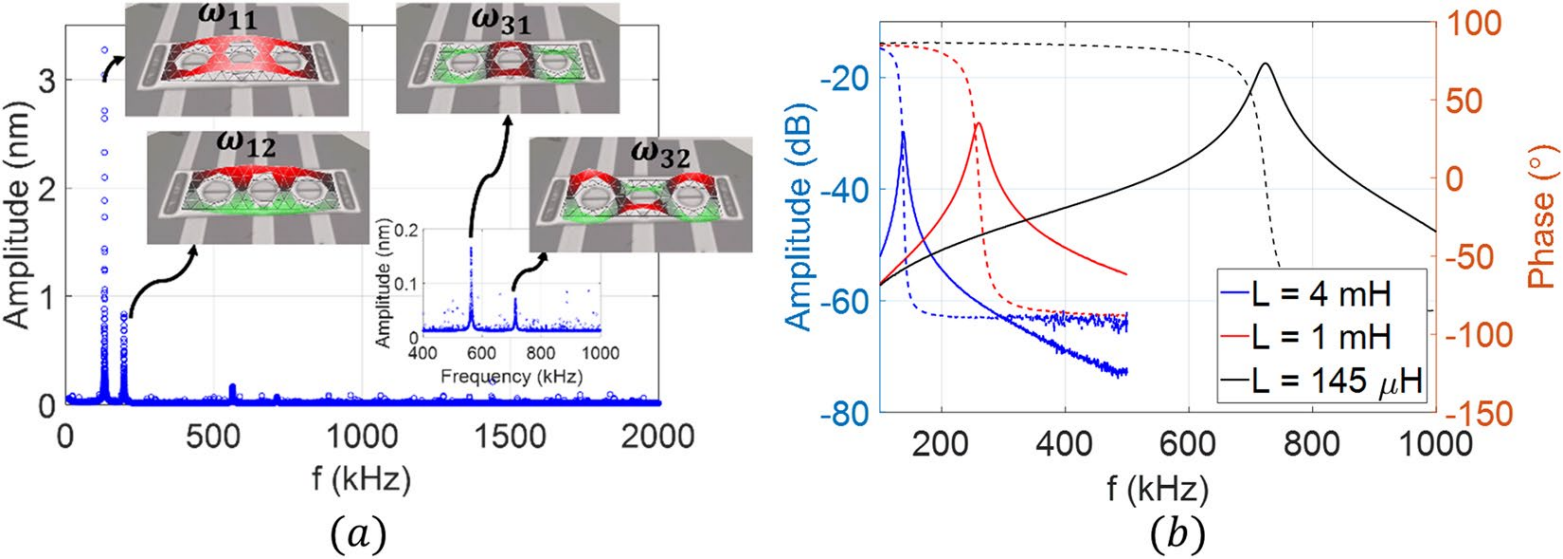
(**a**) Frequency response of the microplate to a white noise excitation at *V*_*DC*_ = *10* *V*, *V*_*AC*_ = *15* *V*, and at chamber pressure *P* = *4 mTorr*. Insets: the corresponding mode shapes acquired by recording the response at different points along the microplate surface using a laser Doppler vibrometer. (**b**) The frequency response of the RLC tank circuit for different inductance values at *V*_*AC*_ = *0* *dBm (316* *mV)* and *V*_*DC*_ = *0* *V*. Transmitted power (solid), phase (dashed).

**Figure 5:**
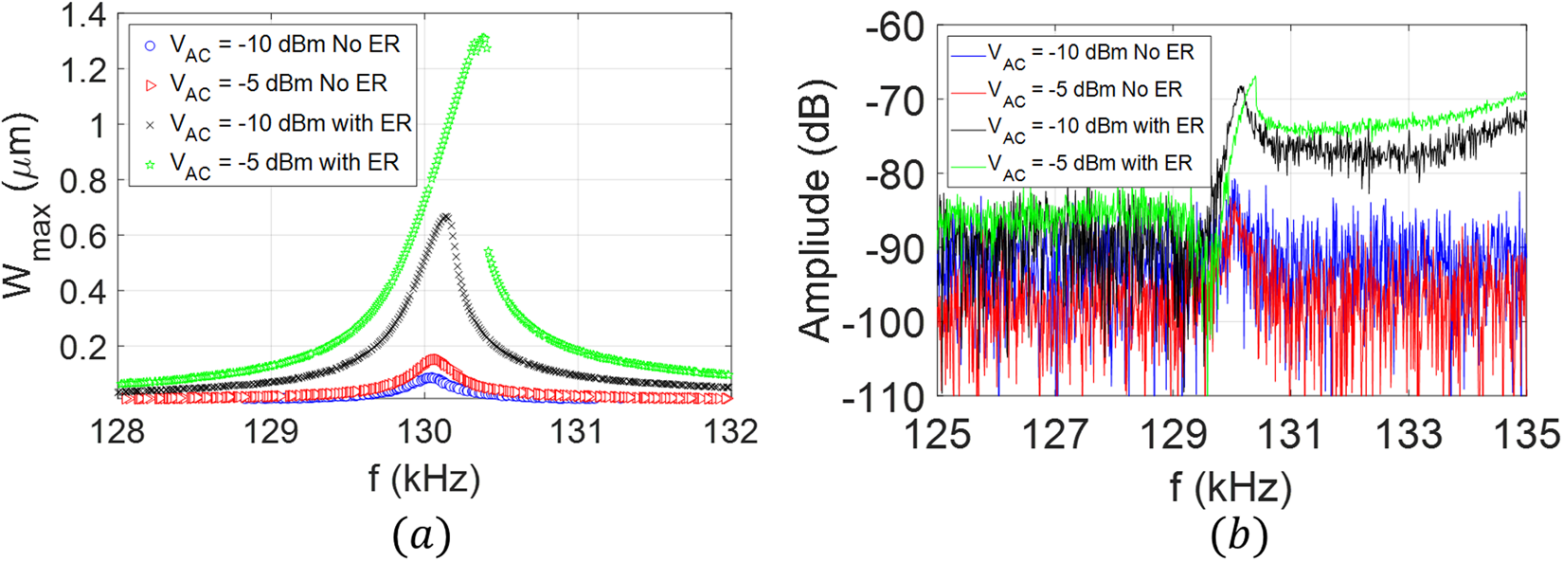
Frequency response of the microplate, for the case *f*_*m*_= *f*_*LC*_, near the first out of plane mode of vibration *ω*_*11*_ due to the activation (ER) and deactivation (NO ER) of the electrical resonance at *V*_*DC*_ = *25* *V and two different AC voltages; V*_*AC*_ = *−10 dBm (99.9* *mV) and V*_*AC*_ = *−5 dBm (177.8* *mV)*. (**a**) Laser Doppler vibrometer measurements, (**b**) network analyzer measurements.

For the initial characterization, we actuate the microplate with a white noise signal while recording the amplitude at different points along the device surface. Figure 4a shows the measured frequency response and the corresponding mode shapes. In this work, we exploit the *LC* tank concept to actuate the first mode at *ω*_*11*_ = *131.2* *kHz* and the higher order mode *ω*_*32*_ = *712.6* *kHz*. Using the direct excitation scheme at *V*_*AC*_ = *0* *dBm (316* *mV)*, we measure the frequency response of the electrical resonance circuit for different inductance values, as shown in Fig. 4b. The circuit is composed of the external inductor *L*, the microplate capacitance *C*_*0*_, the parasitic capacitance from the external cables *C*_*p*_, and the resistance of the circuit *R*. As shown in Fig. 4b, the electrical resonance values at *L*_*1*_ = *4* *mH* is *f*_*e1*_ = *138* *kHz*, and at *L*_*2*_ = *145* *µH* is *f*_*e2*_ = *723.5* *kHz*, which are approximately equal to the mechanical resonance near the first mode *ω*_*11*_ and the higher order mode *ω*_*32*_, respectively. Exact matching of the electrical and mechanical resonances is a challenging task and limited by the availability of tunable inductor with fine steps. Using the experimental setup demonstrated in Fig. 3, without the spectrum analyzer and the second AC source, *V*_*AC2*_, we sweep the excitation frequency around the mode of interest while simultaneously recording the response from the laser Doppler Vibrometer and the network analyzer. As shown in Fig. 5a, by connecting *L*_*1*_, the electrical resonance gets activated near the first vibrational mode frequency of the microplate. Thus, the actuation voltage across the MEMS device is amplified, hence, the maximum amplitude of vibration, *W*_*max*_, increases by eight times compared to the off-resonance case for the same input voltages. The figure also shows that a slight increase in the input voltage drives the resonator into the nonlinear regime (hardening). Figure 5b shows the network analyzer data, which demonstrate the advantage of the proposed technique in elevating the output current above the noise level and detecting the resonance frequency of the resonator compared with very low signal-to-noise ratio in the case of no electrical resonance (no ER).

**Figure 6:**
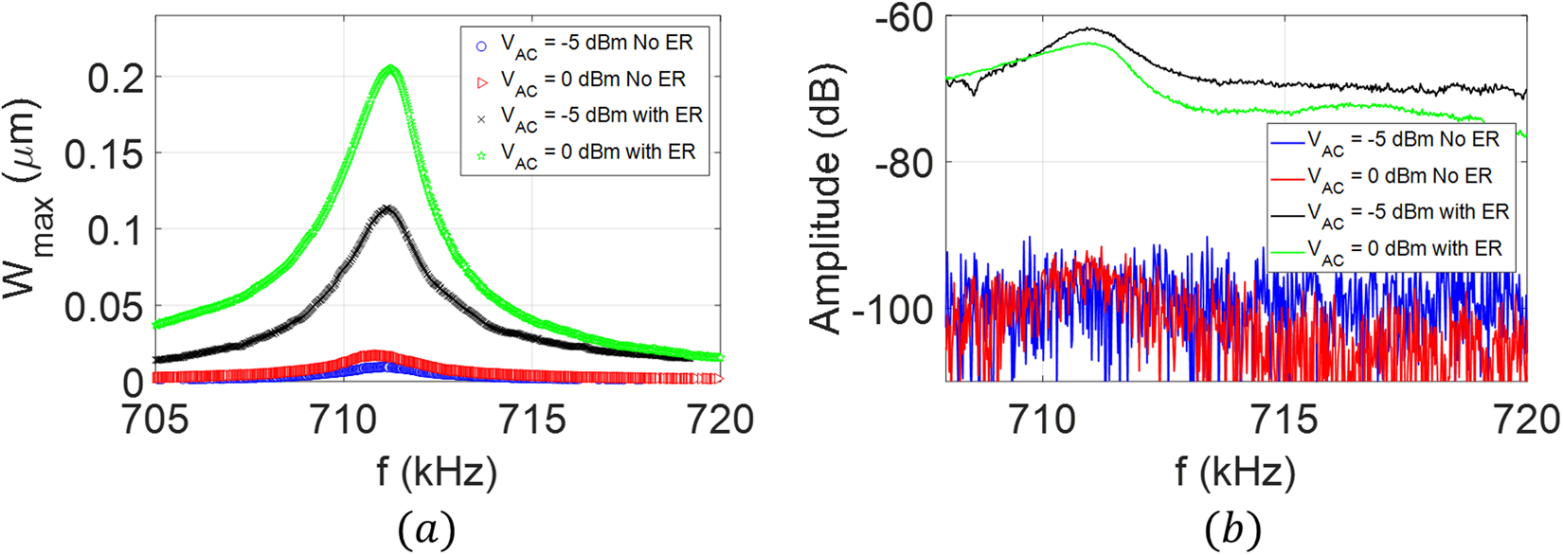
Frequency response of the microplate, for the case *f*_*m*_ = *f*_*LC*_, near the higher order mode of vibration *ω*_*32*_ due to the activation (with ER) and deactivation (No ER) of the electrical resonance at *V*_*DC*_ = *50* *V and two different AC voltages; V*_*AC*_ = *−5 dBm (177.8* *mV) and V*_*AC*_ = *0* *dBm (316.18* *mV)*. (**a**) Laser Doppler vibrometer measurements, (**b**) network analyzer measurements.

Next, *L*_*1*_ is replaced with *L*_*2*_ to allow for simultaneous activation of electrical resonance and the mode *ω*_*32*_. Figure 6a shows the results near the higher order mode at which 12 times amplification factor is reported. Note that in the case of deactivated electrical resonance, the available voltage is not enough to actuate the resonator, and the output motional current is buried in the noise as shown by the blue curve in Fig. 6b. Even increasing the input signal to *V*_*AC*_ = *0* *dBm (316* *mV)* did not reveal a clear resonance peak. One can note that the reported vibration amplitudes amplifications obtained by laser measurements in Figs 5(a) and 6(a) are different from those obtained electrically in Figs 5(b) and 6(b). This is due to the imperfect background signal subtraction (the post-processing of transmission signal magnitude) in the electrical measurement results.

### Case 2

**Figure 7:**
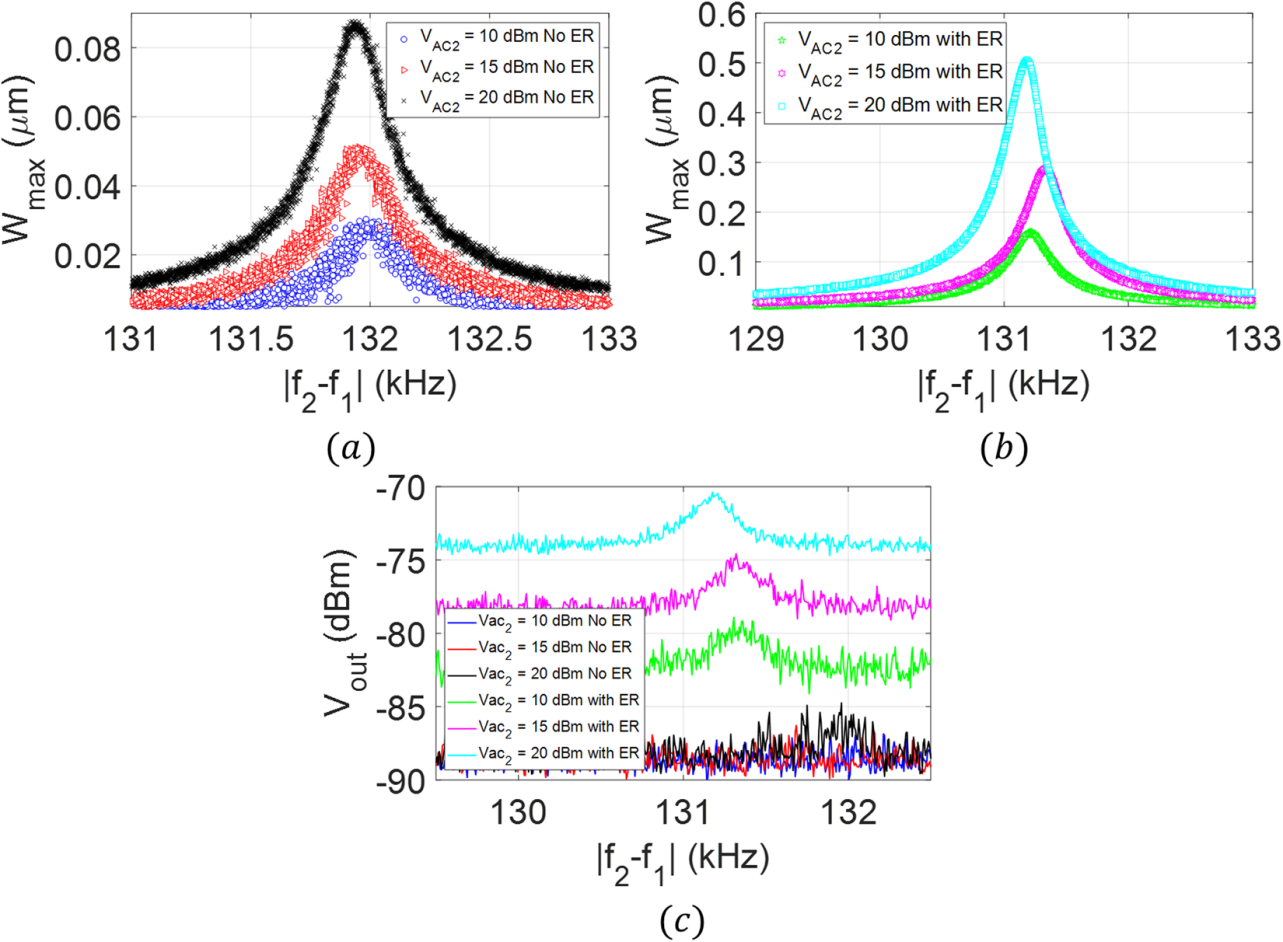
Frequency response of the microplate near the first mode of vibration due to activation (with ER) and deactivation (No ER) of the electrical resonance. The mixed excitation scheme (Fig. 1(b)) is utilized to characterize the microplate at *V*_*DC*_ = *25* *V, V*_*AC1*_ = *10* *dBm (1* *V), and three different AC voltages values of the second source; V*_*AC2*_ = *10* *dBm (1* *V), V*_*AC2*_ = *15* *dBm (1.778* *V), and V*_*AC2*_ = *20* *dBm (3.162* *V)*. (**a**) Laser Doppler vibrometer measurements without the electrical resonance (No ER), (**b**) laser Doppler vibrometer measurements with the electrical resonance (With ER), (**c**) spectrum analyzer measurements.

To demonstrate the flexibility of the proposed technique and to overcome the requirement of matching the electrical and mechanical resonances, we utilize the mixing excitation scheme, Fig. 3. We select inductor *L* = *220 µH* such that the corresponding electrical resonance *f*_*e*_ = *632* *kHz* is far from the mechanical resonance frequencies reported in Fig. 4a. The frequency of the first source *v*_*AC*1_ is tuned at the electrical resonance, *f*_*1*_ = *632* *kHz* while the frequency of the second source *v*_*AC*2_ is swept such that the combinational frequency of difference type *(f*_*2*_ − *f*_*1*_*)* is swept around the first mode of vibration *ω*_*11*_ = *131.2* *kHz*. As shown in Fig. 7a,b, employing the electrical resonance increased the maximum amplitude of vibration by a factor of 6. The spectrum analyzer results, Fig. 7c, demonstrate the significance of the proposed technique in raising the response signal above the noise level compared with noisy response without the electrical resonance.

### Case 3

**Figure 8:**
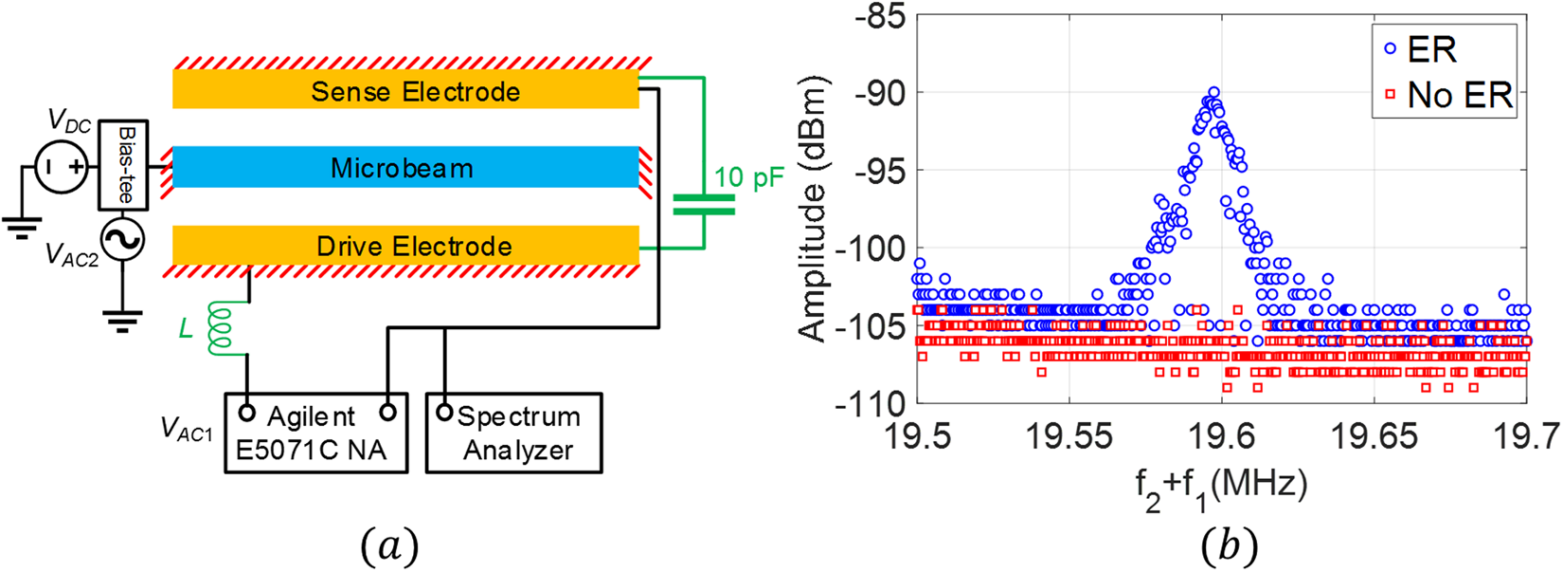
(**a**) The experimental setup utilized to characterize the nanobeam. (**b**) Spectrum analyzer measurements of the nanobeam frequency response near the first mode of vibration, due to the activation and deactivation of the electrical resonance using the mixed frequency excitation scheme.

Finally, we demonstrate the potential of the proposed technique to actuate a nanostructure. To this end, we use the *LC* tank circuit to activate the fundamental mode of a clamped-clamped nanobeam resonator shown in Fig. 2a. As shown in Fig. 8a, we employ the mixing excitation technique to characterize the nanobeam. To form the electrical resonant circuit, we connect a *10* *pF* external capacitor between the sense and drive electrode and an inductor between the drive electrode and the network analyzer such that the electrical resonance is at *f*_*e*_ = *297* *kHz*. The first source frequency is fixed at the electrical resonance frequency *f*_*1*_ = *f*_*e*_ = *297* *kHz*. The second source frequency, *f*_*2*_, is swept to search for the condition when *f*_*1*_ + *f*_*2*_ = *f*_*m*_ = *19.6* *MHz*. *V*_*AC1*_ is fixed at *−5 dBm (177.8* *mV)* and *V*_*AC2*_ is fixed at *23* *dBm (4.466* *V)*. The DC voltage is fixed at *50* *V*. The spectrum analyzer results, Fig. 8b, show the ability of the technique in revealing the resonance frequency of the nanobeam at *19.6* *MHz* compared with no response in the case of deactivated electrical resonance. The reported results demonstrate the importance of this technique in revealing the resonance frequency of nanobeam and increasing the quality factor, which enhances the sensitivity of resonator-based sensors.

## Conclusions

We show a simple technique based on *LC* tank resonant circuit to amplify the electrostatic voltage without using active electronics to efficiently actuate MEMS/NEMS resonators. Several case studies have been presented to show the effectiveness, simplicity, and flexibility of this technique to activate higher order modes of a microplate and the fundamental mode of vibration of a nanobeam. By matching the electrical resonance frequency with the microplate higher order mode of vibration, twelve times amplitude amplification is reported using the same input voltages. Employing this technique eliminated the need of bulky active amplifiers that need external power supply to operate them. Also, active amplifiers are limited by their low gain at high frequencies. To demonstrate the flexibility of the method and overcome the requirement of matching the electrical and mechanical resonance frequency, we employed the mixing excitation technique, where theoretically any inductor value can be utilized. However, using small inductor values with a low resistance value results into a higher quality factor and hence higher voltage gain. Also, we demonstrated the effectiveness of this technique in activating nanoresonators and improving the signal to noise ratio. The amplified electrical actuation force facilitates the exploration of nonlinear phenomena, such as parametric resonances, subharmonic and superharmonic resonances, and higher order modes of stiff nanoresonators, which require high actuation voltages that are usually not available or attainable. The proposed technique can be also extended to amplify the forcing of other actuation mechanism, such as piezoelectric and magnetic.
